# Intrathecal Nicardipine for Severe Intractable Reversible Cerebral Vasoconstriction Syndrome: A Novel Case Report

**DOI:** 10.7759/cureus.42269

**Published:** 2023-07-21

**Authors:** Colin Wakefield, Brendon Ngo, Stanislav Naydin, Rudy Rahme, Mandy Binning

**Affiliations:** 1 Medical School, Drexel University College of Medicine, Philadelphia, USA; 2 Interventional Neuroradiology, Drexel University College of Medicine, Philadelphia, USA; 3 Neurosurgery, Neurosurgeons of New Jersey, Ridgewood, USA; 4 Neuroscience, Drexel University College of Medicine, Philadelphia, USA; 5 Neurological Surgery, Global Neuroscience Institute, Philadelphia, USA

**Keywords:** medical management, cerebral blood flow, new technique, angiogram, vasospasm intervention

## Abstract

Reversible cerebral vasoconstriction syndrome (RCVS) is a poorly understood but increasingly recognized entity, likely multifactorial in nature and characterized by diffuse cerebral vasospasm that presents as sudden, intense, and fluctuating headaches. Due to insufficient evidence, there is currently no consensus on RCVS treatment guidelines. However, nicardipine, an L-type calcium channel blocker, may prove effective in RCVS treatment because of its ability to penetrate the blood-brain barrier. We report the concomitant use of intrathecal (IT) nicardipine and continuous intraarterial (IA) nicardipine infusion via microcatheter placed in the intracranial circulation for the treatment of a 58-year-old female with severe refractory RCVS. On presentation, this patient was noted to have a non-traumatic non-aneurysmal subarachnoid hemorrhage secondary to RCVS. Initially managed with oral verapamil, she later developed refractory symptomatic vasoconstriction requiring multiple angiograms for spasmolysis via balloon angioplasty and IA nicardipine. Due to the refractory nature of her spasm despite the IA therapy, we decided to attempt intrathecal nicardipine, starting at 4 mg q12 h via an external ventricular drain. This dose was escalated to 4 mg q6 h. The patient stabilized for 24 h but again decompensated, requiring continuous IA spasmolysis via a microcatheter placed in the left middle cerebral artery and left for continuous IA nicardipine infusion (5 mg/h). The patient showed slow incremental improvement clinically and a decrease in vasospasms on imaging, ultimately suffering minimal stroke burden. This patient's hospital course demonstrates that nicardipine, administered intrathecally or intraarterially, could be beneficial in select patients with refractory RCVS as a means of minimizing repeat angiography/angioplasty. Further studies are needed to better define a treatment paradigm for these patients.

## Introduction

Reversible cerebral vasoconstriction syndrome (RCVS) is an increasingly recognized entity characterized by the development of segmental cerebral artery narrowing and dilation that commonly resolves spontaneously in three months. RCVS often presents as a severe “thunderclap” headache with or without focal neurological symptoms [1]. The course of RCVS is often benign, with an estimated 4% of patients left with severe disabilities [2]. However, in patients with severe presentations, transient ischemic attacks, cerebral infarctions, leukoencephalopathy, and seizures are reported sequelae [3].

RCVS-induced subarachnoid hemorrhage (SAH) is radiographically distinguishable from aneurysmal SAH due to bleeding localized to the cortex, diffuse segmental vasoconstriction implicating arteries removed from the site of bleeding, and no evidence of a ruptured aneurysm [4]. Treatment modalities for RCVS have not been fully established, as there is sparse literature. Currently, oral and intraarterial (IA) calcium channel blockers, balloon angioplasty, and milrinone have been used [1]. The often-used calcium channel blocker nimodipine has no proven effect on the time course of cerebral vasoconstriction or hemorrhagic and ischemic complications [4]. Nicardipine, another type of L-type calcium channel blocker, may prove effective in RCVS treatment because of its ability to penetrate the blood-brain barrier. However, to date, few case reports have discussed the use of intrathecal (IT) or continuous IA administration of nicardipine for the treatment of severe RCVS [1,5-8]. In this paper, we present the case of a 58-year-old female who presented with refractory RCVS, non-responsive to oral calcium channel blocker, ultimately requiring concomitant IT and continuous IA nicardipine for treatment.

## Case presentation

A 58-year-old female presented to a local emergency department (ED) with complaints of sudden-onset severe headache and associated nausea and vomiting. The patient took over-the-counter non-steroidal anti-inflammatory drugs with no relief. She then developed left arm tingling and mouth tingling.

On physical examination, the patient was drowsy but oriented to person, place, and time, and following simple commands. Her speech was dysarthric. No motor deficits were appreciated. A non-contrast head CT revealed a Hunt-Hess grade 3 SAH. The patient was admitted for neurosurgical evaluation.

**Figure 1:**
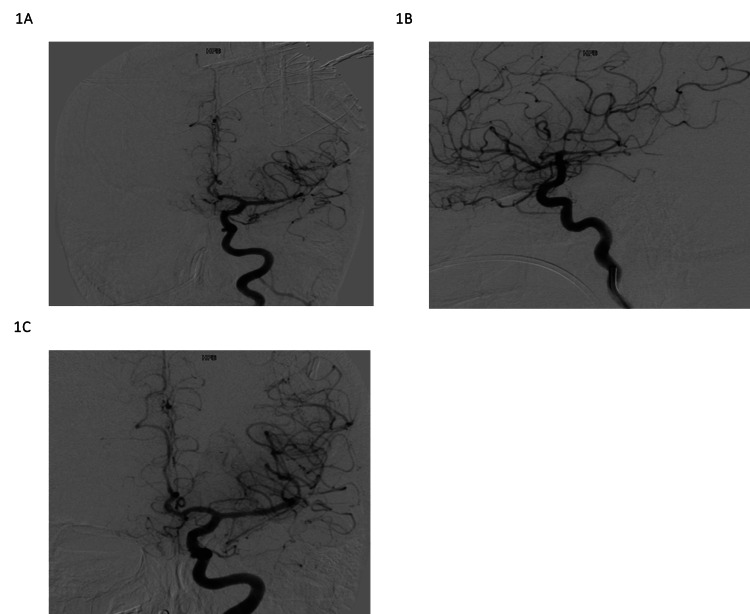
Angiography (A) Initial angiogram demonstrating non-aneurysmal SAH with widespread vasoconstriction correlating with RCVS diagnosis. (B)* *Angiogram on day 6 post-SAH demonstrating refractory vasospasm after multiple previous angiograms of balloon angioplasty and intraarterial nicardipine injections. (C)* *Angiogram on bleed day 9 showing an absence of vasospasm, correlating with improvement in the patient’s clinical condition. SAH, subarachnoid hemorrhage; RCVS, reversible cerebral vasoconstriction syndrome.

A six-vessel diagnostic cerebral angiogram revealed no vascular lesions. However, there was evidence of diffuse spasms in the left middle cerebral artery (L MCA) and in the anterior cerebral artery (ACA) distributions likely representing RCVS or vasculitis (Figure 1A). A negative vasculitis panel later substantiated the diagnosis of RCVS. The following day the patient was drowsy but neurologically intact, capable of walking without assistance and talking. However, on bleed day 3, she began deteriorating with hours-long periods of pronounced hemiplegia and aphasia consistent with worsening spasms. EEG was negative for seizures, and she underwent a repeat diagnostic angiogram with IA nicardipine and resolution of the vasospasm. The patient improved clinically. However, she deteriorated again the next day, and a diagnostic cerebral angiogram showed recurrent severe L MCA vasospasm. IA nicardipine was administered with partial response. A balloon angioplasty of the left M1 was then performed with complete resolution of the spasm and significant improvement of the distal perfusion. Due to the recurrence of symptoms on bleed days 5, 6, and 7, the patient underwent subsequent diagnostic angiography which showed recurrence of the spasm, treated with IA nicardipine in the internal carotid artery (ICA)/MCA/ACA distributions leading to complete resolution of the spasm after each procedure (Figure 1B). However, despite the initial improvement, the patient clinically worsened within hours of the IA treatment requiring further intervention. Continuous EEG did not reveal seizure activity, reaffirming an RCVS mechanism for deterioration.

On bleed day 8, the decision was made to place an extraventricular drain (EVD) to begin IT nicardipine infusions at 4 mg q12 h, mirroring established protocol for the treatment of refractory vasospasms in the setting of aneurysmal SAH [9]. However, the patient only remained stable for 24 hours before again decompensating. On bleed day 8, the patient underwent another cerebral angiogram and an SL10 Microcatheter (Stryker Excelsior® SL-10® Microcatheter, Fremont, California) microcatheter was placed in the L MCA and left in place for continuous IA nicardipine infusion (5 mg/h). Concomitantly, we increased the IT dose to 4mg q6 h. Angiograms on bleed days 9 and 12 later showed no vasospasms (Figure 1C). On bleed day 16, the patient's EVD was removed and nicardipine treatment was discontinued. She was continued on verapamil and slowly began regaining neurological function. She remained in the ICU requiring tracheostomy and percutaneous endoscopic gastrostomy (PEG) tube placement. On the last follow-up, she is continuing to improve, conversational, and moving all extremities except for a slight weakness in the left arm.

## Discussion

RCVS is a poorly understood etiology of cerebral vasospasm. It often presents with a thunderclap headache due to segmental cerebral artery narrowing and dilation. The majority of cases have a mild to moderate disease course with spontaneous resolution. However, some patients experience SAH and ischemic changes as a result of the vasospasm, causing significant long-term neurologic impairment. This case report discusses a patient with a severe presentation of RCVS who initially presented as an SAH.

Refractory vasospasms can occur following a multitude of etiologies including vasculitis, aneurysmal SAH, and RCVS. The current treatment paradigms in these circumstances include daily angiograms and IA therapy. These procedures, specifically when repeated in sick patients, can carry a significant risk of complications including but not limited to intracranial hemorrhage, access site hematomas, thromboembolic events, infection, contrast reactions, kidney damage, and anesthetic complications [10]. Most importantly, as seen in our patient, daily angiography/angioplasty with IA calcium channel blocker infusions can sometimes fall short and do not prevent the recurrence of vasospasm. Therefore, IT and continuous IA microcatheter infusions could be considered as routes of nicardipine administration to definitively decrease vasospasm burden. The risk of lifelong sequelae from EVD or microcatheter placement in an intensive care setting should be considered against the risks of continued widespread cerebral ischemia.

IT nicardipine infusions q6 via an EVD or intraarterially via a microcatheter provide a safer and more definitive alternative treatment than repeat angiography for refractory vasospasm. The EVD is commonly placed following intracerebral hemorrhage to monitor and control intracerebral pressure; therefore, the use of IT infusion poses minimal additional risk for infection or hemorrhage to the patient. A microcatheter, likewise, can be placed during angiography. In patients requiring multiple rounds of angiography, the benefit provided by microcatheter placement during an initial procedure should be weighed against the risk of complications from additional procedures. In the field of neurocritical care, continuous microcatheter infusions are commonly used as a refractory treatment modality. Microcatheter infusions of thrombolytics are currently used to treat cerebral venous thrombosis refractory to initial endovascular intervention [11]. Similarly, continuous IA microcatheter infusion of nimodipine is a rescue treatment for refractory cerebral vasospasm following aneurysmal SAH (aSAH) [12]. Indwelling microcatheter infusions do carry a degree of risks such as hemodynamic instability from systemic vasodilator effect, bloodstream infections, elevations in intracranial pressure (ICP) due to vasodilation of cerebral vasculature, and thromboembolic events. However, the potential burden of such complications can be mitigated through proper precautions likely already used by neurointensive care teams, such as sterile technique and ICP monitoring. To prevent thromboembolic events following IA microcatheter placement, the literature suggests prophylactic anticoagulation [12]. As such the patient discussed in this case report was anticoagulated using a continuous heparin drip.

IT administration of nicardipine mitigates the impact of systemic vasodilation on cerebral perfusion. Calcium channel blockers administered systemically increase the risk of diffuse vasodilation, further compromising oxygenation to end organs such as the brain. However, delivery directly to the cerebral vasculature via IT administration minimizes this risk of hypotension/hypoperfusion [1]. Such an approach also circumvents dosage complications related to drug metabolism and blood-brain barrier diffusion, which arise with oral or intravenous administration. This was an important consideration in our patient, as she had a prior history of hypotension and continued failure of systemic calcium channel blockers, which created a serious risk of compounding RCVS-induced ischemic damage. Our treatment approach was adopted based on the evidence provided by prior case studies describing the successful treatment of refractory vasospasm following aSAH with IT nicardipine. A review by Hafeez et al. demonstrated that IT nicardipine in an aSAH patient mitigates the symptomatic and angiographic burden of refractory vasospasm [9]. The use of IT nicardipine to treat refractory RCVS has only been reported by one other group, in which the patient also failed oral calcium channel blockers in a similar fashion to the patient described in this case report [6]. Therefore, we report the second successful case of IT nicardipine treatment for refractory RCVS, but further investigation is needed to establish efficacy.

## Conclusions

Nicardipine administered intrathecally through an EVD and continuously intraarterially via a microcatheter for the treatment of refractory RCVS vasospasm has been sparsely discussed. We build on the existing fund of knowledge and illustrate that IT/IA nicardipine could be considered for refractory vasospasms to decrease repeat angiography/angioplasty burden and the risk of cerebral hypoperfusion. Further studies are needed to determine the efficacy and safety of continuous IA administration of the neuro-vasculature-specific calcium channel blocker, nicardipine, in RCVS patients.
